# B-cell-guided strategy for SARS-CoV2 vaccination after autologous stem cell transplantation for B-cell lymphoma — a case report

**DOI:** 10.1007/s00277-022-04935-3

**Published:** 2022-08-03

**Authors:** Ben-Niklas Baermann, Paul Jäger, Guido Kobbe

**Affiliations:** Onkologie Und Klinische Immunologie, Klinik Für Haematologie, Universitätsklinikum Düsseldorf, Moorenstr. 5, 40223 Düsseldorf, Germany

Dear Editor,

Since the beginning of the COVID-19 pandemic, recipients of hematopoietic stem stell transplantation (HSCT) are challenging high-risk for critical infections with SARS-CoV2 [1]. After successful implementation of vaccination in healthy individuals, the immunization strategy for HSCT recipients has been discussed due to divergent immune responses in this population. B-cell lymphomas have been identified as an important risk factor for insufficient serologic response to vaccination, especially in patients with recent B-cell-directed therapy, e.g., BTK inhibitors and CD20-directed antibodies [2].

So far recommendations for vaccination after autologous HSCT (autoHSCT) rely on time intervals to transplant. We here report on cellular immune reconstitution and associated serologic response to SARS-CoV2 vaccination in a 54-year-old male patient with DLBCL after autoHSCT.

He suffered from late relapse of DLBCL in 2020. After three cycles of rituximab-based immune chemotherapy and documented complete remission, high-dose chemotherapy with R-TEAM and autoHSCT was performed in September 2020. We observed normal hematopoietic reconstitution in the early phase and until last observation in November 2021, the patient remained in complete remission.

The patient received two doses of BNT162b2 for basic immunization (BI) seven months after HSCT. Prior to first vaccination, absolute T-helper-cell count had almost reached normal levels with 441 cells/µl, but nearly total B-cell depletion was still present. In August and September 2021, two booster vaccinations with BNT162b2 were administered in an interval of six weeks. Before booster vaccination, a significant and steady increase in the peripheral CD19 + -B-cell count could be identified. There was no evidence of SARS-CoV2-infection prior to HSCT or vaccination.

**Fig. 1:**
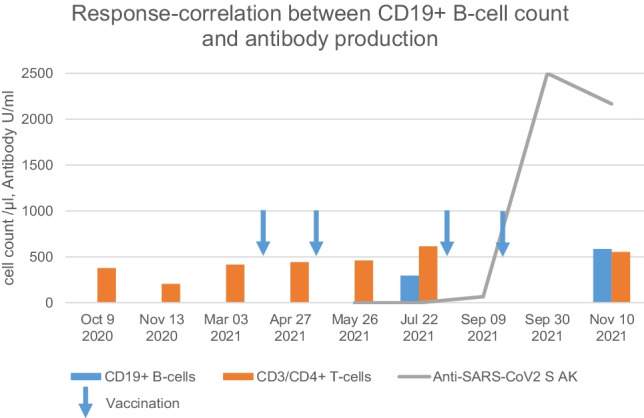
Response-correlation between CD19 + B-cell count and antibody production. SARS-CoV2 S AK, SARS CoV2 spike protein antibody

BI did not result in significant antibody production although the recommended interval to autoHSCT was respected. Between day + 251 and day + 308 (July 2021), there was a sudden increase of peripheral B-cells from 3 to 295/µl. Following a first BNT162b2 booster in August 2021, the titer of Anti-SARS-CoV-2-S increased from < 0.4 to over 60U/ml one month later and to > 2500U/ml after a second booster which was given in September 2021. Two months later titers remained stable, concomitant with normal CD19 + B-cell counts in peripheral blood (Fig. 1).

Vaccination of vulnerable individuals has greatly reduced the threat of SARS-CoV2 infection. Its side effects are reported to be mild to moderate and short-term, except extremely rare but serious complications such as myocarditis and thrombotic thrombocytopenia [3–5]. Therefore, vaccination timing must be adapted to the individual patient’s cellular immune status.

We report on the importance of B-cell recovery after CD20-directed therapy and HSCT serving as a parameter to decide on timepoint especially for booster vaccinations. Even after CD4 + -T-cell reconstitution BI didn’t result in any serologic response. Only a sudden and prominent increase in the peripheral B-cell count months later paved the way for measurable antibody production in this patient, emphasizing the importance of B-cell reconstitution for successful vaccination. T-cell and innate immunity are also important to avoid severe COVID-19 as patients with autoimmune diseases receiving B-cell-depleting therapy generally show successful recovery from COVID-19 [6].

However, humoral immune responses represent one important column of virus defense, which cannot be achieved without a functional B-cell compartment. We therefore recommend to guide vaccination after autoHSCT not by fixed time intervals but by cellular immune reconstitution. Vaccination may start as early as T-cell reconstitution after autoHSCT is observed. As patients who have received B-cell depleting antibodies before autoHSCT may, at the same time, have delayed B-cell recovery, anti-virus-titers may be low or even absent following a first vaccination round. Repeated booster vaccinations at the time of B-cell recovery should therefore be applied in order to achieve high antibody responses. This may be true not only for SARS-CoV2 vaccines but also for other vaccines routinely applied after autoHSCT.
